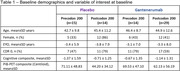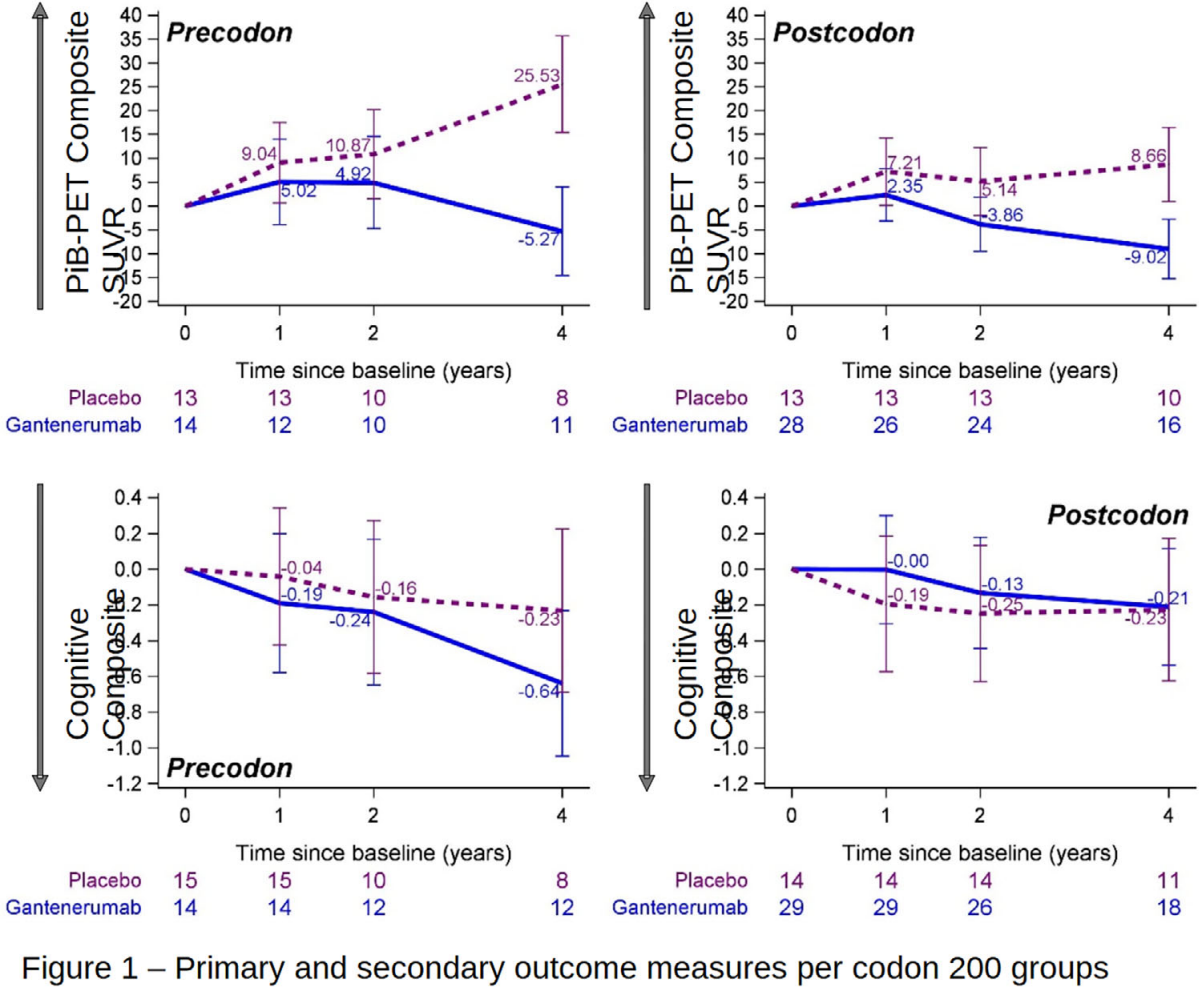# Modeling the influence of presenilin‐1 mutation position on primary outcomes of a gantenerumab trial: findings from the DIAN‐TU‐001 study

**DOI:** 10.1002/alz.095570

**Published:** 2025-01-09

**Authors:** Austin A. McCullough, Yuchen Cao, Charles D. Chen, Brian A. Gordon, Carlos Cruchaga, Alison M. Goate, Jason J. Hassenstab, Laura Ibanez, Yan Li, Richard J. Perrin, Alan E. Renton, Chengjie Xiong, Stephen Salloway, Jorge J. Llibre‐Guerra, David B. Clifford, Eric McDade, Randall J. Bateman, Tammie L.S. Benzinger, Guoqiao Wang, Nelly Joseph‐Mathurin, DIAN study team

**Affiliations:** ^1^ Washington University in St. Louis School of Medicine, St. Louis, MO USA; ^2^ Washington University School of Medicine in St. Louis, St. Louis, MO USA; ^3^ Massachusetts General Hospital, Harvard Medical School, Boston, MA USA; ^4^ Washington University School of Medicine, St. Louis, MO USA; ^5^ Washington University School of Medicine, Saint Louis, MO USA; ^6^ Knight Alzheimer’s Disease Research Center, Washington University, St Louis, MO USA; ^7^ Icahn School of Medicine at Mount Sinai, New York, NY USA; ^8^ Hope Center for Neurological Disorders, St. Louis, MO USA; ^9^ Washington University in St. Louis, School of Medicine, St. Louis, MO USA; ^10^ Knight Alzheimer’s Disease Research Center, St. Louis, MO USA; ^11^ Alpert Medical School of Brown University, Providence, RI USA; ^12^ Butler Hospital, Providence, RI USA; ^13^ Hope Center for Neurological Disorders, Washington University School of Medicine, St. Louis, MO USA; ^14^ Hope Center for Neurological Disorders, Washington University in St. Louis, St. Louis, MO USA; ^15^ Mallinckrodt Institute of Radiology, Washington University in St. Louis, St. Louis, MO USA; ^16^ Knight Alzheimer Disease Research Center, St. Louis, MO USA

## Abstract

**Background:**

In autosomal dominant Alzheimer disease (ADAD), the position of a pathogenic genetic variant within the presenilin‐1 (*PSEN1*) coding sequence influences how amyloid‐beta accumulates and how dementia progresses (Joseph‐Mathurin et al, 2024). In the first trial of anti‐amyloid monoclonal antibodies in individuals at risk for ADAD (Dominantly Inherited Alzheimer Network Trials Unit study 1 [DIAN‐TU‐001]), gantenerumab demonstrated target engagement but did not meet its primary clinical endpoint (Salloway et al, 2021). Whether the distribution of pathogenic genetic variants within the cohort may have influenced the trial results remains unclear. Here, we investigated the primary outcomes from DIAN‐TU‐001 as a function of mutation position.

**Method:**

We evaluated 72 participants with a *PSEN1* mutation who completed the DIAN‐TU‐001 study under the placebo (n = 29) and the gantenerumab (n = 43) arms. Both arms were grouped by the *PSEN1* mutation position relative to codon 200 as precodon‐200 and postcodon‐200 (Table 1). Mixed models for repeated measures were adjusted for sex, baseline Clinical Dementia Rating^®^ (CDR^®^) scores, and baseline EYO and estimated the effect of *PSEN1* mutation position on the mean change from baseline PiB‐PET composite and baseline cognitive composite, as imaging and clinical outcomes, respectively. Significance of differences in least squares (ls) means on year 4 between pre‐codon 200 and post‐codon 200 mutation groups were calculated.

**Result:**

Within each codon group, compared to placebo, the treated group had significant changes in PiB‐PET but not in cognitive composite (Figure 1). Placebo precodon‐200 group showed significantly faster amyloid accumulation rates (PiB PET composite SUVR) through year 4 than the placebo postcodon‐200 group (ls means diff. *P*‐value = 0.0065). However, no difference was observed between precodon‐200 and postcodon‐200 gantenerumab groups (ls means diff. *P*‐value = 0.45) through year 4. Difference in cognitive composite precodon‐200 vs. postcodon‐200 placebo groups was not significant (ls means diff. *P*‐value = 0.98). Differences in cognitive composite groups between treated groups trended toward significance (ls means diff. *P*‐value = 0.05).

**Conclusion:**

These preliminary results show the potential influence of *PSEN1* mutation position on the outcome of a monoclonal antibody targeting amyloid plaque removal. Further investigation into the potential effects of specific mutation type on trial results is warranted.